# Complete Genome Sequence of the Thermophilic Polychlorinated Biphenyl Degrader *Geobacillus* sp. Strain JF8 (NBRC 109937)

**DOI:** 10.1128/genomeA.01213-13

**Published:** 2014-01-23

**Authors:** Masaki Shintani, Yoshiyuki Ohtsubo, Kohei Fukuda, Akira Hosoyama, Shoko Ohji, Atsushi Yamazoe, Nobuyuki Fujita, Yuji Nagata, Masataka Tsuda, Takashi Hatta, Kazuhide Kimbara

**Affiliations:** a Department of Applied Chemistry and Biochemical Engineering, Graduate School of Engineering, Shizuoka University, Hamamatsu, Japan; b Department of Environmental Life Sciences, Graduate School of Life Sciences, Tohoku University, Sendai, Japan; c Biological Resource Center, National Institute of Technology and Evaluation, Tokyo, Japan; d Department of Biomedical Engineering, Graduate School of Engineering, Okayama University of Science, Okayama, Japan

## Abstract

*Geobacillus* sp. strain JF8 (NBRC 109937) utilizes biphenyl and naphthalene as sole carbon sources and degrades polychlorinated biphenyl (PCB) at 60°C. Here, we report the complete nucleotide sequence of the JF8 genome (a 3,446,630-bp chromosome and a 39,678-bp plasmid). JF8 has the smallest genome among the known PCB degraders.

## GENOME ANNOUNCEMENT

*Geobacillus* sp. strain JF8 (NBRC 109937; JCM 19604), isolated from bark compost in Okayama, Japan, can degrade biphenyl and/or polychlorinated biphenyls (PCBs) (1). JF8 can grow at temperatures up to 75°C, but not at 30°C, and its optimum growth temperature is 60°C (1). JF8 can grow on several aromatic compounds, including biphenyl, *p*-chlorobiphenyl, benzoate, naphthalene, and salicylic acid (1). JF8 possesses an approximately 40-kb plasmid, pBt40, which carries biphenyl-degradative genes, including *bphDA1A2BC* (2). DNA sequence information from the JF8 chromosome has been limited to a single operon comprising *nahHLOM*-*mocB*-*nahC*, which includes two genes for extradiol dioxygenase, those encoding 1,2-dihydroxynaphthalene (*nahC*) and catechol (*nahH*) (3).

The genome sequence of JF8 was determined using a combined strategy of GS FLX Titanium (Roche) and HiSeq 1000 (Illumina) technologies. Two different types of libraries were constructed for sequencing, a standard fragment library for GS FLX Titanium and a mate pair library (average pair distance of 6.0 kb) for HiSeq 1000. For Illumina mate pair reads, pairs of reads whose lengths exceeded 50 bp were subjected to 21-mer based filtering using ShortReadManager. In the filtering, the part of the read consisting of 21-mer that appeared only once in the total Illumina reads was removed. The assembly was performed using Newbler v 2.8 software (4). A total of 132,287,318 base sequences (38-fold genome coverage; 221,984 reads) from the GS FLX Titanium system and 285,388,041 base sequences (82-fold genome coverage; 2,508,532 reads) from the HiSeq 1000 system were used for assembly. We obtained 3 scaffolds and 57 large contigs (>500 bp). The finishing was facilitated by *in silico* analyses using our two original software tools, GenoFinisher and AceFileViewer (5) (http://www.ige.tohoku.ac.jp/joho/gf_e/), in which the DNA sequences of each copy of repeats were precisely determined. The finished sequence was confirmed by FinishChecker, which is an accessory tool of GenoFinisher. We also amplified all repeat regions by PCR and sequenced the PCR products to confirm the accuracy of the *in silico* finishing (we corrected only one mistake of a deletion of 348 bp). The genome of JF8 consists of a circular chromosome (3,446,630 bp; 52% G+C) and a circular plasmid named pBt40 (39,678 bp; 46% G+C).

Sequence annotation was performed using the NCBI Prokaryotic Genomes Automatic Annotation Pipeline (http://www.ncbi.nlm.nih.gov/genome/annotation_prok/), and the resulting annotation was manually inspected with respect to the start codon positions using the Microbial Genome Annotation Pipeline (http://www.migap.org/) as well as another annotation support tool of GenomeMatcher (6). The JF8 chromosome has 3,551 coding sequences, 10 sets of rRNA genes, and 88 tRNA genes. JF8 has the smallest genome (3.49 Mb) among the completely sequenced *Geobacillus* strains (3.51 to 3.91 Mb) (http://www.ncbi.nlm.nih.gov/genome/browse/) and also among the PCB degraders, such as *Acidovorax* sp. strain KKS102 (5.20 Mb) (5), *Burkholderia xenovorans* LB400 (9.73 Mb) (7), *Novopshingobium aromaticivorans* DSM 12444 (4.23 Mb) (GenBank accession no. NC_007794, NC_009426, and NC_009427), *Pseudomonas pseudoalcaligenes* KF707 (5.96 Mb) (8), *Rhodococcus jostii* RHA1 (9.70 Mb) (9), and *Rhodococcus* sp. strain R04 (9.13 Mb) (10).

### Nucleotide sequence accession numbers.

The nucleotide sequences of the JF8 chromosome and pBt40 were deposited in the NCBI GenBank database under accession numbers CP006254 and CP006255, respectively.
